# Enlarged colony housing promotes linear progression of subchondral bone remodeling in joint instability rat models

**DOI:** 10.3389/fphys.2023.1232416

**Published:** 2024-01-08

**Authors:** Stephanie Menges, Kerstin Kleinschmidt-Dörr, Christian Brenneis

**Affiliations:** ^1^ Merck Healthcare KGaA, Darmstadt, Germany; ^2^ Merck KGaA, Darmstadt, Germany

**Keywords:** osteoarthritis, subchondral bone, rats, joint instability, colony housing

## Abstract

**Objective:** Osteoarthritis (OA) is a disease with high prevalence and an unmet medical need for disease modifying treatments. In rat models, OA-like subchondral bone and cartilage changes can be induced by instability surgery with different severity levels. Factors which determine structural changes additionally comprise the study duration and activity-impacted joint loading.

**Methods:** A medial meniscal tear (MMT) or anterior cruciate ligament transection with partial meniscectomy (ACLT+pMx) was induced unilaterally in rats housed in a rat colony cage (RCC), allowing high activity levels including jumping and stair climbing. In parallel, ACLT+pMx rats were housed in Type IV cages. The time course of OA-related changes was investigated at 4, 8, 12, and 16 weeks after surgery by micro-CT, gait analysis and joint diameter measurements.

**Results:** Gait disturbance was observed after 2 weeks and to a similar extend in all models. The increase in ipsilateral joint diameters peaked after 2 weeks and were more pronounced after ACLT+pMx compared to MMT-surgery, but independent of housing. Micro-CT analysis revealed that increases in osseous tibial width were most distinct after ACLT+pMx in RCC and progressed continuously until week sixteen. In contrast, osseous tibial width of ipsilateral joints in MMT RCC and ACLT+pMx Type IV groups did not increase further after week twelve. In contralateral joints, this parameter was not affected, regardless of the model or caging. However, a significant increase in bone volume fraction and trabecular thickness was observed over time in the femur and tibia of both ipsilateral and contralateral knees. Here, the medial tibial compartment of the operated joint was most affected and linear changes were most pronounced in the ACLT+pMx RCC group.

**Conclusion:** Increased movement of animals in colony cages leads to robust structural changes in subchondral bone after surgically induced joint instability over time, while in regular Type IV housing maximal changes are reached in week twelve. The new insights into the differentiation of the models, particularly with respect to the linear progression of bone changes in ACLT+pMx in the RCC, may be useful for the design of chronic OA-studies within a longer lifespan and therefore supporting the development of novel therapies.

## 1 Introduction

With high prevalence and an unmet medical need for disease modifying treatments, OA ranks as the second leading cause of physical disability globally. It is characterized by a progressive deterioration of multiple joint tissues, including subchondral bone and cartilage, accompanied by chronic pain (McCune et al., 1990; Naredo et al., 2005). Principal risk factors include: age, family history or genetic predisposition, obesity and traumatic events disturbing joint biomechanics, and are frequently accompanied by mild systemic inflammation (Suri et al., 2012).

The complex anatomy and interrelated pathological processes of OA present challenges to clinical diagnosis and pharmaceutical treatment. The avascular environment combined with the dense matrix of cartilage form a barrier, impeding the penetration of drugs and leading to limited bioavailability of administered substances, and therefore new perspectives to overcome the treatment dilemmas of OA are needed (Li et al., 2023). Besides mechanical and biological factors, cartilage and subchondral bone play a critical role in the development and progression of this disease. Throughout adulthood, bone remodeling stands as the principal metabolic process regulating the structure and function of bones. Therefore, a close interplay between osteoclasts, cells that degrade bone, and osteoblasts, cells that synthesize bone, is essential to maintain mineral homeostasis (Kular et al., 2012). Besides those two predominant cell types, other (immune) cells have also been implicated in bone remodeling and related diseases. Osteocytes, a subpopulation of osteoblasts, seem to initiate and regulate the remodeling process that repairs the damaged bone (Raggatt and Partridge, 2010). In addition, Osteomacs as resident tissue macrophages, are suggested to play an essential role in maintaining mature osteoblasts (Chang et al., 2008). Furthermore, osteoimmunology data proves the participation of immune cells in bone homeostasis, e.g., with activated T-cells being implicated in conditions resulting in bone loss (Weitzmann and Pacifici, 2007) and mast cells being connected with bone marrow fibrosis (Lowry et al., 2008). While the precise role of the subchondral bone in the onset and progression of OA is not fully understood, there is increasing evidence that subchondral bone abnormalities, such as bone marrow oedema and the formation of new blood vessels, occur prior to the degeneration of cartilage. An increase in subchondral bone turnover resulting from abnormal mechanical loading, and angiogenesis and nerve innervation promoting the degradation of cartilage and chondrocyte hypertrophy both expedite bone remodeling. Ultimately, the abnormal mechanical support resulting from subchondral bone sclerosis triggers cartilage destruction (Hu et al., 2021).

It is well known, that physical activity impacts OA-progression in humans (Kraus et al., 2019). The protective or harmful effect of physical activity on OA-progression may be determined by the intensity and quality of the activity, as well as the specific pathophysiological conditions present during active phases. Low prevalence regarding OA was found in weight-bearing joints of active people (Ponzio et al., 2018). In addition, meta-analysis revealed improvements in pain and joint function achieved by certain exercises at the early stages of OA (Goh et al., 2019). On the other hand, excessive overloading of specific joint regions after traumatic injuries can promote catabolic processes in the joint (Wu et al., 2000) and initiate joint degeneration and OA (Andriacchi and Mündermann, 2006).

To elicit OA in rats, subchondral bone and cartilage changes can be induced by instability surgery, with varying levels of severity (Bendele, 2001; Hayami et al., 2006; Lampropoulou-Adamidou et al., 2014; McCoy, 2015; Kuyinu et al., 2016). The intensity of changes is influenced by several factors, including the duration and the degree of joint loading affected by activity (Iijima et al., 2015; Huesa et al., 2022).

Hayami et al. found structural bone changes starting to occur approximately 2 weeks after surgery followed by significant increases in subchondral bone volume after 4–6 weeks in rats operated with the ACLT+MMx model (Hayami et al., 2006). These findings were in accordance to other studies, where ACLT+pMMx induced osteophyte formation after 4–6 weeks (Gerwin et al., 2010). For MMT-models in the rat, degenerative structural changes were described to occur at 3–6 weeks post-surgery (Bendele, 2001; Janusz et al., 2002). In all published results, either the housing cage was not described in particular, or rats were housed in standard cages, therefore it is assumed that increased locomotion was not possible as a factor regarding structural changes. Not only in human OA-progression, as mentioned above, increased mobility options play a central role. In rats, high-impact exercises were also found to contribute to increased OA-progression and severity (Williams et al., 1982; Abbud Lozoya and Kouri Flores, 2000; Appleton et al., 2007), while slight or moderate exercise had a beneficial effect on chondral lesions in the ACLT-model (Galois et al., 2004).

In order to represent the correlation between OA and locomotion more accurately, we designed the Rat Colony Cage (RCC). This caging system provides strong social interaction and a complex environment, leading to highly enriched housing that has proven to be of benefit to animal welfare (Brenneis et al., 2017). The RCC can accommodate a colony of up to 48 rats on four functionally differentiated levels, interconnected by jump holes and staircases allowing vertical movements between floors.

Previously we could show that this novel way of housing, which induces higher spontaneous activity in rats leading to frequent jumps and stair walks, aligns individual behavioral adaptations such as gait disturbance and increases structural bone and cartilage changes in three different models of traumatic joint instability (Brenneis et al., 2020; Chang et al., 2021). One limitation of this study was, that structural endpoints were analyzed at a late timepoint of 20 weeks only. To investigate the therapeutic benefit of potential DMOADs it is important to start the treatment at a time of disease onset and to examine structural endpoints before reaching the plateau phase. In order to choose the ideal necropsy time point covering the linear progressive phase, depending on the model, and housing condition used, we characterized the time course of functional and structural endpoints at 4, 8, 12, and 16 weeks following MMT or ACLT+pMx in RCC and ACLT+pMx in Type IV housing in this study.

## 2 Materials and methods

### 2.1 Animals and housing conditions

Male Lister Hooded (Crl:LIS) outbred SPF rats (8–10 weeks with a weight of 150–220 g) were purchased from Charles River Laboratories (Sulzfeld, Germany). At the breeder, rats were housed in conventional polycarbonate cages (595 mm × 380 mm × 200 mm high; floor area, 1,820 cm^2^) in groups of 5 animals per cage. On arrival at our facility, 24 rats were housed in the same type IV cages in pairs of 3 and the other 96 rats were directly transferred to colony housing cages. Here 48 Lister Hooded rats were housed together, later covering both surgical models (ACLT+pMx, MMT) and all four lifespans (4, 8, 12, and 16 weeks). Distribution ensured equal average weight of rats in different housing conditions. For both housing types, single animals represent the experimental unit. For detailed husbandry conditions see Brenneis et al. (2017). Day-night-rhythm was inverted with red light during 6 a.m. and 6 p.m. All procedures were reviewed and approved by the animal protection authorities of the local district government (approval DA 4/1019 Instability-OA longitudinal, Regional Authorities of Hesse, Darmstadt, Germany).

Exclusion criteria were applied, when, e.g., an animal had to be euthanized due to animal welfare reasons by defined humane endpoints. Those criteria were listed in the animal proposal upfront and approved by the authorities. One rat (group MMT RCC 12w) was found dead in the cage after 8 weeks in the study and pathology revealed tumor cell embolus in the right atrium of the heart. Another rat (group ACLT Type IV 16w) died under anesthesia during surgery at the beginning of the experiment. Due to a severe swollen left ankle joint on the hind paw, one rat (group MMT RCC 16w) had to be euthanized 13 weeks post-surgery.

### 2.2 Surgically induced OA in rats

Surgery was performed after 4 weeks of habituation in their respective caging system and animals were allocated to groups in a randomized manner. To model chronic OA in consequence of different degrees of joint destabilization longitudinally, joint instability was induced with necropsy after 4, 8, 12 and 16 weeks in either RCC housing (*n* = 12 rats/timepoint with ACLT+pMx or MMT surgery) or in Type IV cages (*n* = 6 rats/timepoint with ACLT+pMx surgery).

Details regarding methods for ACLT+pMx, MMT-surgeries, CatWalk tests and joint diameter measurements can be found in supplementary methods listed in Brenneis et al. (2020). In brief, procedures were as follows:

ACLT+pMx: A skin incision was made on the medial side of the right joint, parallel to the patella. The joint capsule was opened, and the anterior cruciate ligament was transected using a hook. The anterior meniscotibial tendon was dissected with a scalpel, and the medial meniscus was partially removed, leaving approximately 10% in the joint to avoid damage.

MMT: A skin incision was made on the medial side of the right joint, parallel to the patella. A longitudinal section was made between the quadriceps tendon and medial collateral ligament. The collateral ligament was loosened with forceps and ligated at the location of the meniscus. The medial meniscus was then completely dissected at the smallest area of the collateral ligament.

### 2.3 In-life outcome measures

Catwalk gait analysis (CatWalk XT 10.0 system, Wageningen, Netherlands) was performed in all rats to evaluate the change of gait characteristics in weeks 0, 2, 4, 6, 8, 10, 12, 14 and 16 after surgery. To train the rats for the test upfront, raspberry syrup was used, and during the actual testing, rats voluntarily walked three times per time point and were visually monitored with boundary surface optics between 7 a.m. and 3 p.m. Animals were tested sequentially, and groups were randomized. Runs with at least 3 sequential steps were analyzed, and the relative print length of the ipsilateral hind paw over the contralateral hind paw was calculated as a measure of gait disturbance.

Joint swellings were measured with a caliper on weeks 0, 2, 4, 8, 12 and 16 after surgery. Rats were anesthetized with 3%–4.5% of isoflurane and put into lateral recumbency with the leg slightly stretched. An electronic caliper (type “quick mini”, Mitutoyo, Neuss, Germany) was placed to the middle of the joint space (the widest location of the joint) and closed, while the leg was fixed with hand on the foot. Then, the leg was released from the finger and moved up and down. Once the measurement stabilized within ±0.1 mm, it was recorded. Joint diameters were calculated as % increase of the diameter of the operated (right) knee joint compared to contralateral.

CatWalk, joint diameter measurements and imaging analysis were conducted in a blinded way to ensure operator-independent results.

### 2.4 Micro-computed tomography

After reaching the defined endpoints of 4, 8, 12 or 16 weeks in the study, rats were euthanized by exsanguination, specifically, terminal cardiac blood collection under surgical depth of isoflurane anesthesia. The joints were tied with gauze to a plastic angle with the geometrical form of a frustum and around 140°, to prevent deformation during fixation in 4% paraformaldehyde (PFA, Merck, Darmstadt, Germany; in 1x phosphate buffered saline (PBS) pH 7.4, Gibco, Waltham, USA) for at least 5 days to reach full fixation. Prior to micro-CT imaging, the joints were rinsed in 1x PBS for 2 h to remove residual PFA and individually packed into small resealable bags (70 × 100 × 0.05 mm bag LDPE zipper, VWR, Radnor, USA). Specimens were scanned using a SkyScan 1176 micro-CT scanner (Bruker, Kontich, Belgium) with an x-ray source of 65 kV/384 μA, a pixel size of 17.60 µm and a 1 mm aluminum filter. Cross-sectional slices were generated using NRecon software (version 1.6.9.4, Bruker) with beam hardening at 30%, smoothing at level 3, and ring artifact corrections at level 5 applied and using defined threshold values to distinguish low-density tissue/air from high-density bone (Bouxsein et al., 2010). All data sets were adjusted to anatomical markers with DataViewer software (version 1.5.4, Bruker) in the same manner to ensure uniform analysis of the datasets. Examples of whole joint micro-CT images can be found in Figures 4C–F. Three-dimensional analysis was performed using CTAn software (version 1.17.7.2, Bruker). The mean total cross-sectional tibia area (B.Ar) was analyzed by generating a maximum intensity projection of the tibial plateau (Figures 3B–E). Cylindric (femur) and ellipsoid (tibia) volumes of interest in the center of the weight-bearing trabecular region of the medial and lateral femoral condyles and tibia plateaus were analyzed (VOI femur: 84 pixels in diameter, 25 images inside VOI; VOI tibia: 142 (width) and 185 (height) pixels in diameter, 25 images inside VOI). Individual data points were either depicted as % for bone volume fraction (BV/TV), µm for Tb.Th, µm^2^ for cross sectional bone area or normalized to the contralateral values of each animal and depicted as % contralateral in the plots.

### 2.5 Statistical analysis

Statistical analysis was performed with GraphPad Prism version 7.03. The respective tests used are indicated in the figure legends.

All data are presented as mean ± SEM. XY-graphs were used for body weight gain, joint swelling, and CatWalk. Imaging data from bones were expressed as scatter dot plots.

Outlier testing was performed using the ROUT method provided by GraphPad Prism (coefficient [Q] = 1%; identifies outliers from nonlinear regression). Individual data were excluded if the ROUT method identified a particular data point as a significant outlier.

Before applying statistical tests, data was checked for normal (Gaussian) distribution with Shapiro-Wilk normality test (significance level (alpha) of 0.05).

Multiple comparisons were conducted by looking at the differences in means of each column (timewise comparison/intragroup comparison of timepoints or of RCC-housing (ACLT RCC v. MMT RCC) or in the same surgery model (ACLT RCC vs. ACLT Type IV). Due to clarity of graphs, results of intergroup comparison tests were not indicated in the figures, but results were mentioned in the captions.

**FIGURE 1:**
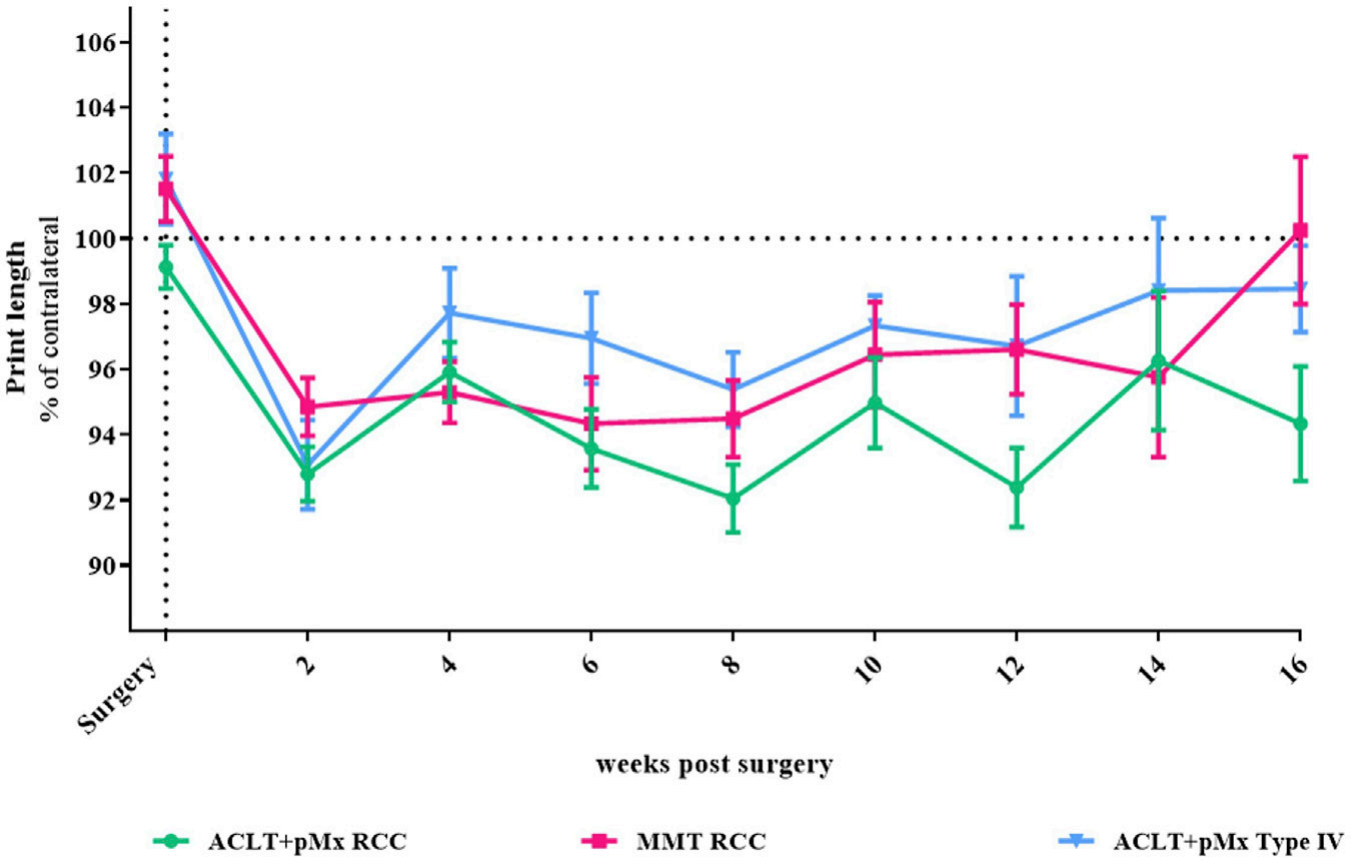
Time course of gait disturbance via CatWalk-analysis; *n* = 5–48; mean ± SEM; dotted horizontal line indicates 100%; two outliers via ROUT method detected and excluded from analysis (ACLT+pMx RCC week 6: 85.26%; ACLT+pMx RCC week 8: 124.46); additional data excluded due to internal CatWalk quality requirements for paw print analysis (*n* = 8); data did not pass Shaprio-Wilk test for normal distribution; no significant differences in mixed-effects model analysis with Šídák’s multiple comparisons test.

**FIGURE 2:**
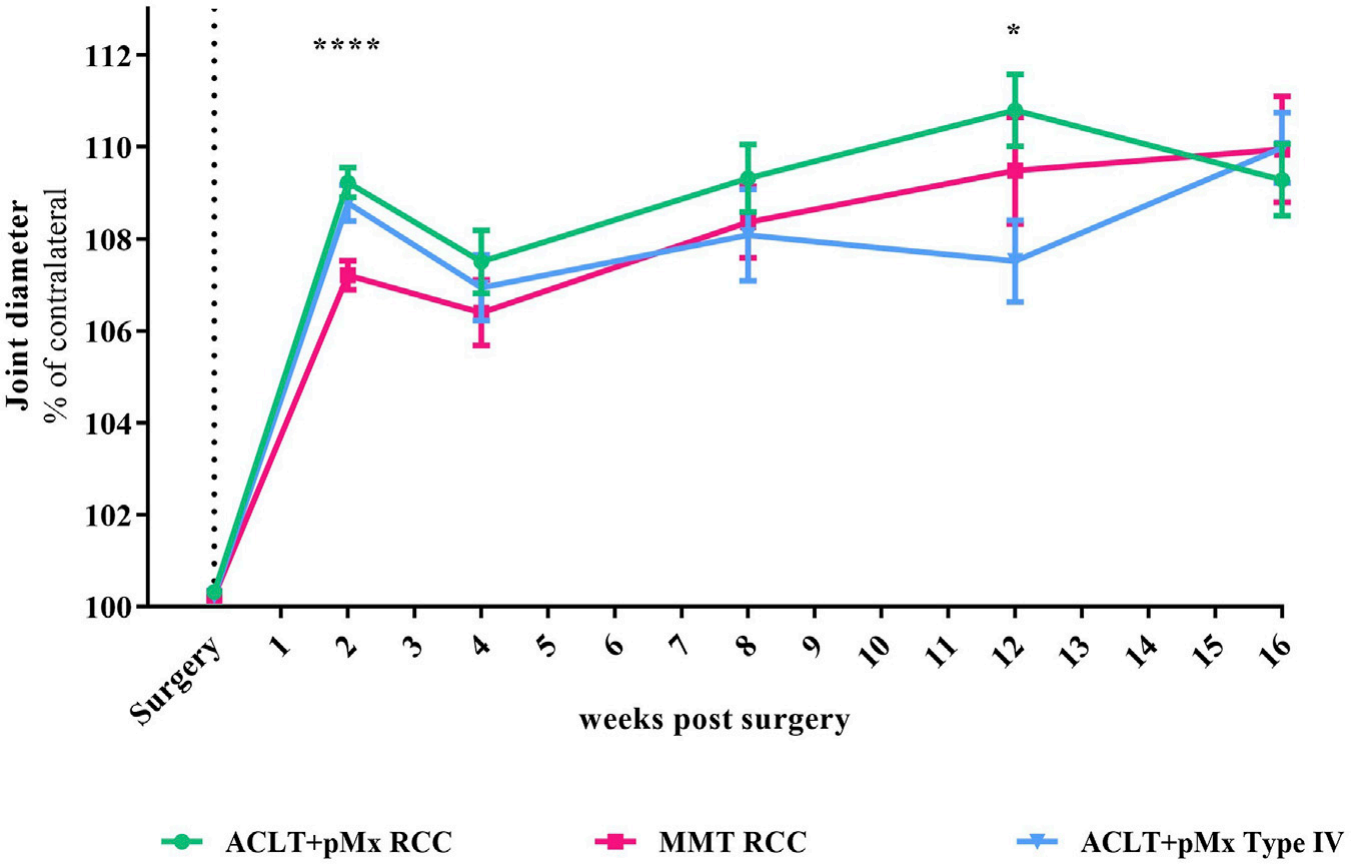
Time course of joint diameter; *n* = 5-48; mean ± SEM; no outliers via ROUT method detected; data did not pass Shaprio-Wilk test for normal distribution; results of mixed-effects model analysis with Šídák’s multiple comparisons test: week 2 ****p < 0.0001 ACLT+pMx RCC vs. MMT RCC; week 12 *p < 0.05 ACLT+pMx RCC vs. ACLT+pMx Type IV.

**FIGURE 3:**
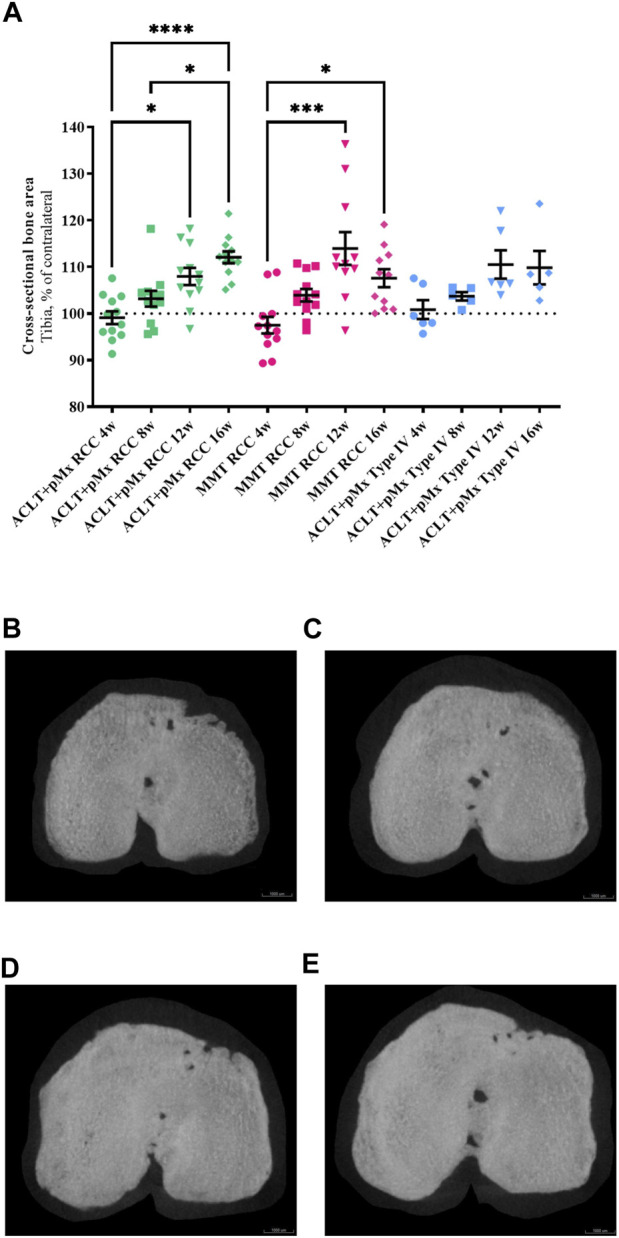
**(A)** Analysis of the cross-sectional bone area of the ipsilateral tibia in comparison to the contralateral tibia; *n* = 11/12 (RCC), *n* = 5/6 (Type IV); mean ± SEM; dotted horizontal line indicates 100%; one outlier via ROUT method detected and excluded (ACLT+pMx Type IV 8w: 121.54%); data did not pass Shaprio-Wilk test for normal distribution; results of Kruskal–Wallis test with Dunn’s multiple comparison test: *p < 0.05 ACLT+pMx RCC 4w vs. ACLT+pMx RCC 12w; *p < 0.05 ACLT+pMx RCC 8w vs. ACLT+pMx RCC 16w; *p < 0.05 MMT RCC 4w vs. MMT RCC 16w; ***p < 0.001 MMT RCC 4w vs. MMT RCC 12w; ****p < 0.0001 ACLT+pMx RCC 4w vs. ACLT+pMx RCC 16w. **(B)** Micro-CT image of the cross-sectional bone area from the ipsilateral tibia of animal no. 80, ACLT+pMx RCC 4w group. Scale bar = 1000 µm. **(C)** Micro-CT image of the cross-sectional bone area from the ipsilateral tibia of animal no. 36, ACLT+pMx RCC 8w group. Scale bar = 1000 µm. **(D)** Micro-CT image of the cross-sectional bone area from the ipsilateral tibia of animal no. 22, ACLT+pMx RCC 12w group. Scale bar = 1000 µm. **(E)** Micro-CT image of the cross-sectional bone area from the ipsilateral tibia of animal no. 32, ACLT+pMx RCC 16w group. Scale bar = 1000 µm.

**FIGURE 4:**
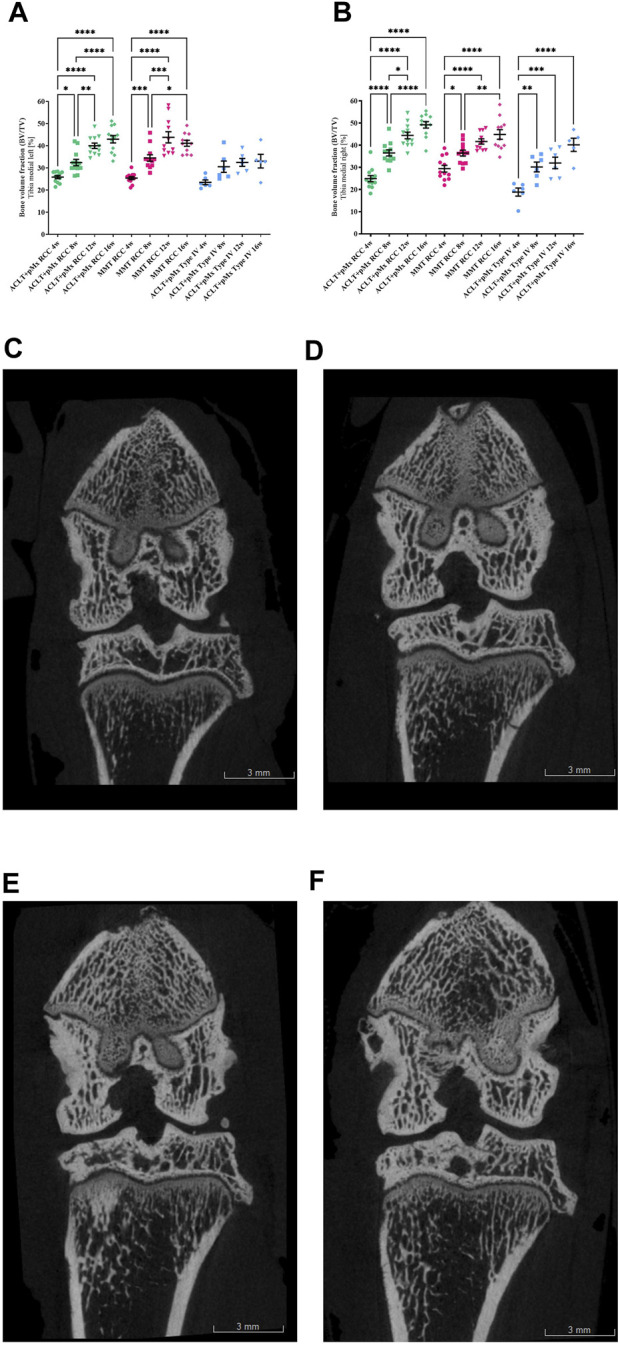
**(A)** Bone volume fraction analysis (BV/TV) of the left medial Tibia; *n* = 11/12 (RCC), *n* = 5/6 (Type IV); mean ± SEM; no outliers via ROUT method detected; data passed Shaprio-Wilk test for normal distribution; results of 1way ANOVA with Šídák’s multiple comparisons test: *p < 0.05 ACLT+pMx RCC 4w vs. ACLT+pMx RCC 8w; *p < 0.05 MMT RCC 8w vs. MMT RCC 16w; **p < 0.01 ACLT+pMx RCC 8w vs. ACLT+pMx RCC 12w; **p < 0.01 ACLT+pMx RCC 16w vs. ACLT+pMx Type IV 16w; ***p < 0.001 MMT RCC 4w vs. MMT RCC 8w; ***p < 0.001 MMT RCC 8w vs. MMT RCC 12w; ****p < 0.0001 ACLT+pMx RCC 4w vs. ACLT+pMx RCC 12w; ****p < 0.0001 ACLT+pMx RCC 4w vs. ACLT+pMx RCC 16w; ****p < 0.0001 ACLT+pMx RCC 8w vs. ACLT+pMx RCC 16w; ****p < 0.0001 MMT RCC 4w vs. MMT RCC 12w; ****p < 0.0001 MMT RCC 4w vs. MMT RCC 16w. **(B)** Bone volume fraction analysis (BV/TV) of the right medial Tibia; *n* = 11/12 (RCC), *n* = 5/6 (Type IV); mean ± SEM; no outliers via ROUT method detected; data passed Shaprio-Wilk test for normal distribution; results of 1way ANOVA with Šídák’s multiple comparisons test: *p < 0.05 ACLT+pMx RCC 8w vs. ACLT+pMx RCC 12w; *p < 0.05 MMT RCC 4w vs. MMT RCC 8w; *p < 0.05 ACLT+pMx RCC 16w vs. ACLT+pMx Type IV 16w; **p < 0.01 MMT RCC 8w vs. MMT RCC 16w; **p < 0.01 ACLT+pMx Type IV 4w vs. ACLT+pMx Type IV 8w; ***p < 0.001 ACLT+pMx Type IV 4w vs. ACLT+pMx Type IV 12w; ***p < 0.001 ACLT+pMx RCC 12w vs. ACLT+pMx Type IV 12w; ****p < 0.0001 ACLT+pMx RCC 4w vs. ACLT+pMx RCC 8w; ****p < 0.0001 ACLT+pMx RCC 4w vs. ACLT+pMx RCC 12w; ****p < 0.0001 ACLT+pMx RCC 4w vs. ACLT+pMx RCC 16w; ****p < 0.0001 ACLT+pMx RCC 8w vs. ACLT+pMx RCC 16w; ****p < 0.0001 MMT RCC 4w vs. MMT RCC 12w; ****p < 0.0001 MMT RCC 4w vs. MMT RCC 16w; ****p < 0.0001 ACLT+pMx Type IV 4w vs. ACLT+pMx Type IV 16w. **(C)** Coronal plane image of a micro-CT-scan of the ipsilateral knee joint of animal no. 80, ACLT+pMx RCC 4w group. Scale bar = 3 mm. **(D)** Coronal plane image of a micro-CT-scan of the ipsilateral knee joint of animal no. 36, ACLT+pMx RCC 8w group. Scale bar = 3 mm. **(E)** Coronal plane image of a micro-CT-scan of the ipsilateral knee joint of animal no. 22, ACLT+pMx RCC 12w group. Scale bar = 3 mm. **(F)** Coronal plane image of micro-CT-scan of the ipsilateral knee joint of animal no. 32, ACLT+pMx RCC 16w group. Scale bar = 3 mm.

**FIGURE 5:**
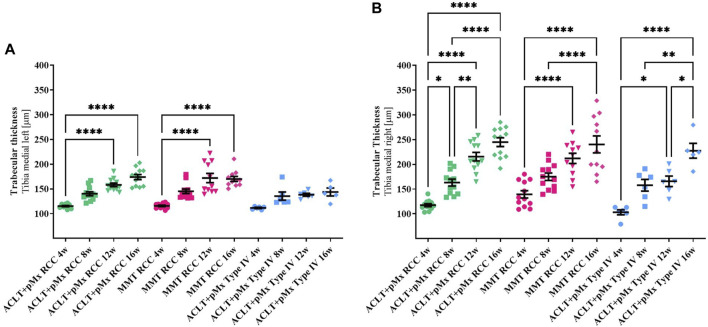
**(A)** Trabecular thickness (Tb.Th) of the left medial Tibia; *n* = 11/12 (RCC), *n* = 5/6 (Type IV); mean ± SEM; no outliers via ROUT method detected; data did not pass Shaprio-Wilk test for normal distribution; results of Kruskal–Wallis test with Dunn’s multiple comparison test: ****p < 0.0001 ACLT+pMx RCC 4w vs. ACLT+pMx RCC 12w; ****p < 0.0001 ACLT+pMx RCC 4w vs. ACLT+pMx RCC 16w; ****p < 0.0001 MMT RCC 4w vs. MMT RCC 12w; ****p < 0.0001 MMT RCC 4w vs. MMT RCC 16w. **(B)** Trabecular thickness (Tb.Th) of the right medial Tibia; *n* = 11/12 (RCC), *n* = 5/6 (Type IV); mean ± SEM; no outliers via ROUT method detected; data passed Shaprio-Wilk test for normal distribution; results of 1way ANOVA with Šídák’s multiple comparisons test: *p < 0.05 ACLT+pMx RCC 4w vs. ACLT+pMx RCC 8w; *p < 0.05 ACLT+pMx Type IV 4w vs. ACLT+pMx Type IV 12w; *p < 0.05 ACLT+pMx Type IV 12w vs. ACLT+pMx Type IV 16w; *p < 0.05 ACLT+pMx RCC 12w vs. ACLT+pMx Type IV 12w; **p < 0.01 ACLT+pMx RCC 8w vs. ACLT+pMx RCC 12w; **p < 0.01 ACLT+pMx Type IV 8w vs. ACLT+pMx Type IV 16w; ****p < 0.0001 ACLT+pMx RCC 4w vs. ACLT+pMx RCC 12w; ****p < 0.0001 ACLT+pMx RCC 4w vs. ACLT+pMx RCC 16w; ****p < 0.0001 ACLT+pMx RCC 8w vs. ACLT+pMx RCC 16w; ****p < 0.0001 MMT RCC 4w vs. MMT RCC 12w; ****p < 0.0001 MMT RCC 4w vs. MMT RCC 16w; ****p < 0.0001 MMT RCC 8w vs. MMT RCC 16w; ****p < 0.0001 ACLT+pMx Type IV 4w vs. ACLT+pMx Type IV 16w.

For time course analysis (xy-graphs, Figures 1, 2), a mixed-effects model analysis with Šídák’s multiple comparison test was applied, as data did not pass Shaprio-Wilk test for normal distribution. For column analysis (Figures 3–5) a Kruskal–Wallis test with Dunn’s multiple comparison test was performed, when data did not pass Shaprio-Wilk test for normal distribution, respectively a 1way ANOVA with Šídák’s multiple comparisons test was conducted for data passing Shaprio-Wilk test for normal distribution.

## 3 Results

### 3.1 CatWalk gait disturbance measurements

In our previous studies we found through CatWalk analysis of spatial and temporal footprint patterns, that the parameter most significantly impacted in the chronic phase of OA-models was the print length (covering the span of the complete paw), which is expressed as a percentage of the contralateral hind paw (Brenneis et al., 2020). Here, 2 weeks after surgery, all three models (ACLT+pMx in RCC or Type IV, MMT RCC) exhibited a comparable level of gait disturbance in relation to the contralateral leg (Figure 1), with trends towards recovery in gait disturbance at later timepoints in MMT RCC and ACLT+pMx groups. Over the course of the study, the levels of print length stayed below their baseline measurements. However, due to high intergroup variabilities, no significant differences between the groups could be observed.

### 3.2 Joint diameter

Besides structural changes like bone remodeling and cartilage loss, symptoms of OA are also associated with joint broadening. By measuring the joint diameter with an electronic caliper, we found an increase of 7%–9% compared to the contralateral leg 2 weeks after surgery (Figure 2). The increase in joint diameter caused by surgery after 2 weeks was significantly more distinct in ACLT+pMx in both housing systems compared to the MMT-model (*p* < 0.0001 for ACLT+pMx RCC vs. MMT RCC). After 12 weeks, a slightly decreased joint swelling was present in the ACLT+pMx Type IV-group compared to ACLT+pMx in RCC-housing (*p* = 0.0327). No difference in joint swelling was visible between all groups after week sixteen.

### 3.3 Micro-computed tomography

#### 3.3.1 Cross-sectional bone area of tibia

As OA progresses, the bones are no longer cushioned by cartilage and friction occurs while the joints are moving. To stabilize its joints, the body responds to this process by increasing the bone mass and density leading to osteophyte formation and enlarged joint surfaces. By analyzing the cross-sectional bone area of the tibia, we found a continuous linear increase in osseous tibial width of operated right joints in ACLT+pMx RCC rats over time (Figure 3A), beginning at week eight with an expansion of 4.1%, and constant linear enlargement of the cross-sectional area of up to 13% until week sixteen. At the same time, no further increase was seen after the twelfth week in MMT RCC and ACLT+pMx Type IV groups. No significant bony expansion of the tibial plateau compared to the contralateral side could be detected after 4 weeks in all models. The cross-sectional bone area of the tibia in both RCC-models at late stage (week twelve and sixteen) was significantly higher when compared to week four. Regardless of model or caging, no linear increase in osseous tibial width was observed in the unoperated contralateral joints (Supplementary Figure S1).

#### 3.3.2 Bone volume fraction (bone volume per tissue volume)

Analysis of the trabecular bone structures revealed a significant increase in BV/TV in all joint areas over time in RCC housing. The medial (Figures 4A, B) and lateral tibia (Supplementary Figures S3, S4) of both the ipsilateral and contralateral legs were affected. In addition, the same effect on BV/TV could be observed in all femoral joint parts (Supplementary Figures S5–S8).

Due to surgical manipulation of the medial meniscus, the medial tibial compartment of the operated joint has the highest risk for increased BV/TV in all three groups.

In the ACLT+pMx RCC group, significant and linear changes were especially pronounced and the bone volume per tissue volume doubled from week four (24.9%) to week sixteen (49.2%) in the medial tibia of the ipsilateral leg. Other joint parts (lateral Tibia, medial & lateral Femur, contralateral legs) revealed progressive increases in the bone volume fraction, as well. Similar significant results could be observed in the MMT-group, but with less linear effects. Also, in Type IV-housed rats, significant changes in BV/TV could mainly be detected in the medial tibia part of the right leg, while in the lateral tibia and lateral and medial femoral parts only slight changes over time were observed. At the earliest timepoint of 4 weeks, effects on the bone volume fraction of the operated leg (Tibia and Femur) appeared strongest in the MMT-model, without reaching significance when compared to ACLT+pMx housed in the same RCC.

In addition, meaningful differences between the two housing systems of RCC and Type IV in the ACLT+pMx model were visible in the medial tibia of both legs at later timepoints.

Furthermore, BV/TV in general appeared higher in RCC-housed rat legs compared to Type IV housing.

#### 3.3.3 Trabecular thickness

In addition to bone volume analysis, trabecular thickness was analyzed as a second imaging indicator for subchondral sclerosis.

A significant increase in Tb.Th in all joint areas was observed over time in the RCC housing when trabecular bone structures were examined. Medial (Figures 5A, B) and lateral (Supplementary Figures S9, S10) tibiae of both ipsilateral and contralateral legs were involved, with the same effect to be observed in the femora (Supplementary Figures S11–S14).

Like seen in the BV/TV-results, the medial compartment of the operated joint was mostly affected by increased Tb.Th in all three groups with linear changes best represented in both RCC-groups. Significant effects on Tb.Th in Type IV-housed rats were found only in the right medial part of the tibia and femur, while the other parts of the joints showed only mild changes over time.

Increases in Tb.Th in lateral parts of both legs appear to be less steep in all three models when comparing the different timepoints to the medial parts.

## 4 Discussion

As a musculoskeletal disorder, OA exhibits a noticeable relationship between the usage of the impacted joint and the progression of the disease with the joints responding via structural remodeling. In rat models, cartilage degradation with subchondral bone remodeling develops over time from increased activity-impacted joint loading after instability surgery. Currently, most rat OA-studies are performed in one-dimensional Type IV cages, despite there being a known association between OA and physical activity. Our RCC encompasses socialization opportunities and multidimensional movement options that promote spontaneous engagement in load-bearing activities to stimulate the rats’ natural behavior (Brenneis et al., 2017; 2020).

Since rats typically exhibit low levels of spontaneous knee degeneration, lesions observed are usually the result of surgical manipulation (Smale et al., 1995; Gerwin et al., 2010). To determine the optimal necropsy time point during the progressive phase, considering the specific model and housing conditions employed, we conducted a study with longitudinal investigation of functional and structural endpoints. We evaluated gait disturbance, joint diameter, and bone remodeling at 4, 8, 12, and 16 weeks following MMT or ACLT+pMx surgery in RCC housing, and with ACLT+pMx in Type IV-housing as comparison. Due to research department’s closure, no histological sections are available for this study. In our previous study we detected reduced cartilage volume in histological evaluations after 20 weeks using the RCC-housing (Brenneis et al., 2020) system, with cartilage mostly affected in the medial tibia area.

By CatWalk analysis we found spontaneous gait disturbance was most noticeable 2 weeks after surgery at a comparable level between all three groups. This symptomatic result regarding early phase-effects was found in our previous experiment, as well (Brenneis et al., 2020). When following the course of the symptoms until week sixteen, the MMT RCC and ACLT+pMx Type IV group showed trends towards improvement in gait disturbance. The recovery in the ACLT+pMx RCC group appears to be less pronounced. Gait disturbance can be interpreted as pain-related compensating behavior, and following a degenerative event, pain occurs as a result of an inflammatory response (Christiansen and Stevens-Lapsley, 2010; Misu et al., 2022). These functional results may therefore give a first indication, that rats from the ACLT+pMx RCC group have prolonged active changes during the course of the study potentially due to cartilage erosion and/or bone remodeling. To demonstrate significant and more conclusive data, higher animal numbers per group would be necessary. Furthermore, alternative methods like dynamic weight-bearing during jumping that reflect more sensitive functional changes in these rat OA-models might add additional insights into symptomatic characterization of OA (Westhof et al., 2021).

In addition to structural alterations such as cartilage loss and subchondral bone remodeling, OA is also characterized by an enlargement of the affected joint. Surgically induced instability triggers compensatory mechanisms that result in increased osteophyte development and subsequent enlargement of the tibial plateau (van der Kraan and van den Berg, 2007). In our study, detected increases in joint diameters at early timepoints (two and four weeks after surgery) were most likely related to soft tissue swelling that might have resulted from the surgical intervention itself and acute inflammatory processes due to cartilage damage (Hayami et al., 2006). This thesis was supported by the analysis of the cross-sectional bony tibia areas of ipsilateral and contralateral legs revealing no structural enlargement measured by micro-CT after 4 weeks (see Figure 3). Cross-sectional bony tibia areas were created by maximum intensity projections of the topview of the tibial plateau. Those projections include the enlargement of all adjacent bone structures in the upper tibia and bony outgrowth at the joint margins, the osteophytes. It is important to bear in mind, that this method does not quantify subchondral bone changes inside the trabecular structures, that start to occur early in OA (Aizah et al., 2021) and were analyzed separately by BV/TV and Tb.Th. In addition, joint swelling of both ACLT+pMx-groups was more distinct compared to the MMT-model after 4 weeks, eventually resulting from heavier interventions during surgery by not only cutting the ACL, but also resecting most parts of the medial meniscus instead of only transecting the medial meniscal ligament (MMT). After 8 weeks, increased joint diameters could be related to bony changes at the joint margins, as corresponding micro-CT-findings revealed enlarged cross-sectional tibial areas at the same timepoint.

Besides the correlation of detected joint swelling and broadening of the bony tibia area starting at week eight in our models, micro-CT analysis revealed further expansion of the cross-sectional ipsilateral tibia until 16 weeks after surgery with most prominent linear effects in the ACLT+pMx-group housed in the RCC. This suggests that the reported joint diameter, measured with a caliper, is not solely influenced by broadening of the tibial plateau. Edema due to joint inflammation may influence the joint diameter, especially in the early phase, as well. High variability at later timepoints, especially in the operated legs in the cross-sectional tibia analysis (week twelve and sixteen, Supplementary Figure S2) supports the hypothesis, that a sufficient number of animals per group is needed to account for the variability in lesion severity (Bendele, 2001).

In addition to enlarged tibial areas, the bone volume fraction (BV/TV) and trabecular thickness (Tb.Th) in the area of the subchondral trabecular bone below the cortical plate significantly increased over time in all parts of the femur and tibia in ipsilateral and contralateral knee joints. Here, the medial tibial compartment of the operated joint was most affected and linear changes were most pronounced in ACLT+pMx RCC. The subchondral trabecular bone is a metabolically active zone (Zhu et al., 2021) which corresponds to mechanical stimuli by regulation of bone mass and architecture (Lanyon, 1996). In Type IV cages, BV/TV, and Tb.Th. had tendencies to be lower in contralateral legs starting from week four on. This effect was covered by the surgery-induced subchondral bone sclerosis in the ipsilateral legs but was still less pronounced when Type IV cage-animals were compared to RCC-housing.

After 4 weeks, we found slightly higher BV/TV and Tb.Th. in MMT compared to ACLT+pMx-operated legs. This corresponds to the literature findings, as published results (Gerwin et al., 2010) postulate the MMT-surgery to result in more severe disease manifestation compared to the ACLT+pMx-model. Nevertheless, joint swelling and cross-sectional tibial areas did not reflect these findings. Over time, we found a more linear progression of OA in the ACLT+pMx model in our colony housing system. Data from later timepoints generated in this study (twelve and sixteen weeks), as well as from our previous experiments after 20 weeks (Brenneis et al., 2020), revealed no increased severity of MMT over ACLT+pMx.

The novelty of this study was to investigate the time course of structural changes after surgically induced OA, with an additional focus on the contribution of housing and the varying degrees of associated spontaneous movements. Physical activity influences the progression of osteoarthritis (OA) in humans, with effects being dependent on the intensity, quality, and specific pathophysiological conditions present during activity (Kraus et al., 2019). Active individuals demonstrate lower prevalence of OA in weight-bearing joints (Ponzio et al., 2018) and certain exercises have been shown to improve pain and joint function in early-stage OA (Goh et al., 2019). However, excessive overloading of specific joint regions following traumatic injuries can trigger catabolic processes (Wu et al., 2000), leading to joint degeneration and OA (Andriacchi and Mündermann, 2006). We found that the progressive increase in physical activity of animals following surgically induced joint instability and housed in colony cages over a period of time results in more substantial structural alterations in the subchondral bone. A number of clinical conditions result from an imbalance in the bone remodeling process which favors either osteoclast or osteoblast activity, with the exact underlying mechanisms remaining largely unclear. In addition, many diseases of bone are associated with an immune component (Raggatt and Partridge, 2010). It was found that resistance training improved bone strength and induced an upregulation of MMP-2 activity and collagen 1, highlighting the importance of exercise for bone health (de Farias Junior et al., 2020). In addition, mechanical conditioning promoted extracellular matrix content and thickness of cell sheets *in vitro* with osteogenic-specific gene expression and matrix-components being upregulated. After implantation of the cell sheets in mice, new bone formation was significantly elevated (Wang et al., 2023). To promote the understanding of the cellular and molecular mechanisms involved in OA-related bone remodeling, investigation of the effects of enlarged colony housing and high locomotion activity on different cell types in the bone microenvironment of OA-models in rats would bear important insights.

To better understand the correlation between OA and locomotion, the Rat Colony Cage (RCC) was designed, providing a complex and enriched housing environment that promotes higher spontaneous activity and aligns with behavioral and structural changes in joint instability models. Besides enhancing animal welfare (Hurst et al., 1999), our findings offer valuable new insights into the distinct characteristics of various models, particularly regarding the linear advancement of bone modifications in the ACLT+pMx group within the colony cage environment. Depending on a specific question or study objective (e.g., to demonstrate a structural benefit of an anticatabolic asset) it may help to achieve an optimal test window and thereby reduce the number of animals required per dose group. As disease progression was characterized in a translational housing system over time, those insights hold potential significance in guiding the design of preclinical OA-studies for therapy development.

## Data Availability

The raw data supporting the conclusion of this article will be made available by the authors, without undue reservation.
