# Supplementary material for: Enlarged colony housing promotes linear progression of subchondral bone remodeling in joint instability rat models
Source: Front Physiol. 2024 Jan 8;14:1232416. doi: 10.3389/fphys.2023.1232416 (PMC10800552; doi:10.3389/fphys.2023.1232416)
Supplement: Supplementary file 1 [file DataSheet1.docx]

Supplementary Material

# Supplementary Figures and Tables

## Supplementary Figures

**
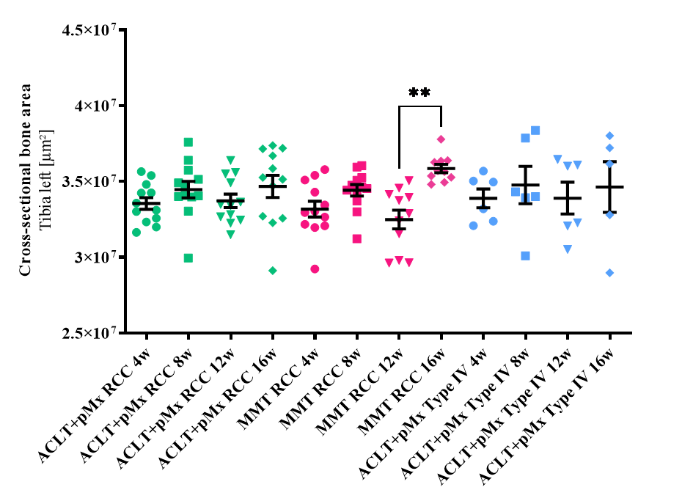
**

**Supplementary Figure 1.** Analysis of the cross-sectional bone area of the left tibia; *n*=11/12 (RCC), *n*=5/6 (Type IV); mean ± SEM; one outlier via ROUT method detected and excluded (MMT RCC 16w: 3.0249x10^7^ µm²); data passed Shaprio-Wilk test for normal distribution; results of 1way ANOVA with Šídák's multiple comparisons test: ***p*<0.01 MMT RCC 12w vs. MMT RCC 16w.


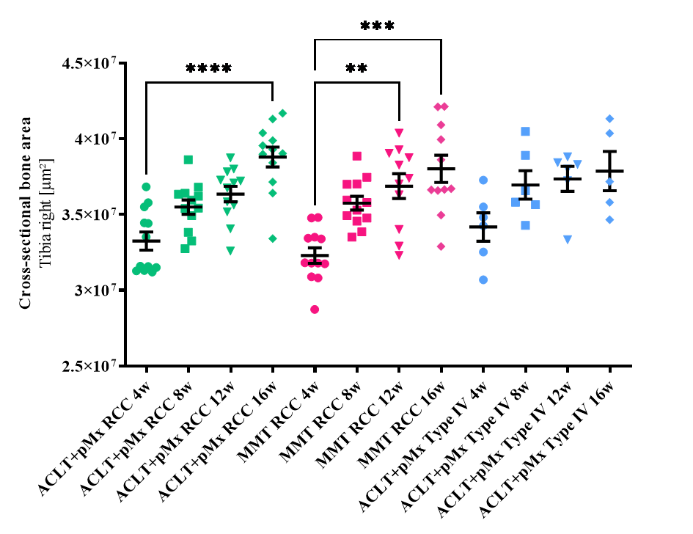


**Supplementary Figure 2.** Analysis of the cross-sectional bone area of the right tibia; *n*=11/12 (RCC), *n*=5/6 (Type IV); mean ± SEM; no outliers via ROUT method detected; data did not pass Shaprio-Wilk test for normal distribution; results of Kruskal-Wallis test with Dunn’s multiple comparison test: ***p*<0.01 MMT RCC 4w vs. MMT RCC 12w; ****p*<0.001 MMT RCC 4w vs. MMT RCC 16w; *****p*<0.0001 ACLT+pMx RCC 4w vs. ACLT+pMx RCC 16w.


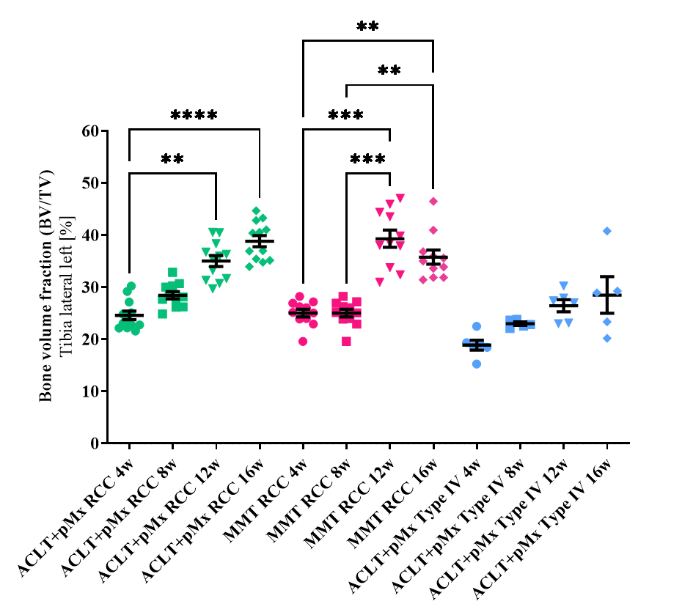


**Supplementary Figure 3.** Bone volume fraction analysis (BV/TV) of the left lateral Tibia; *n*=11/12 (RCC), *n*=5/6 (Type IV); mean ± SEM; four outliers via ROUT method detected (ACLT+pMx RCC 8w: 43.22 %; MMT RCC 4w: 46.15 %; MMT RCC 8w: 46.15 %; ACLT+pMx Type IV 8w: 31.14 %); data did not pass Shaprio-Wilk test for normal distribution; results of Kruskal-Wallis test with Dunn’s multiple comparison test: ***p*<0.01 ACLT+pMx RCC 4w vs. ACLT+pMx RCC 12w; ***p*<0.01 MMT RCC 4w vs. MMT RCC 16w; ***p*<0.01 MMT RCC 8w vs. MMT RCC 16w; ****p*<0.001 MMT RCC 4w vs. MMT RCC 12w; ****p*<0.001 MMT RCC 8w vs. MMT RCC 12w; *****p*<0.0001 ACLT+pMx RCC 4w vs. ACLT+pMx RCC 16w.


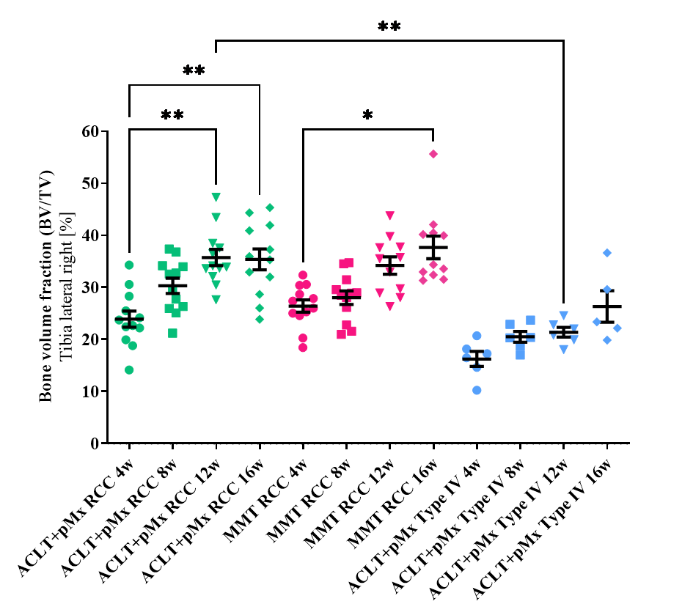


**Supplementary Figure 4.** Bone volume fraction analysis (BV/TV) of the right lateral Tibia; *n*=11/12 (RCC), *n*=5/6 (Type IV); mean ± SEM; no outliers via ROUT method detected; data did not pass Shaprio-Wilk test for normal distribution; results of Kruskal-Wallis test with Dunn’s multiple comparison test: **p*<0.05 MMT RCC 4w vs. MMT RCC 16w; ***p*<0.01 ACLT+pMx RCC 4w vs. ACLT+pMx RCC 12w; ***p*<0.01 ACLT+pMx RCC 4w vs. ACLT+pMx RCC 16w; ***p*<0.01 ACLT+pMx RCC 12w vs. ACLT+pMx Type IV 12w.


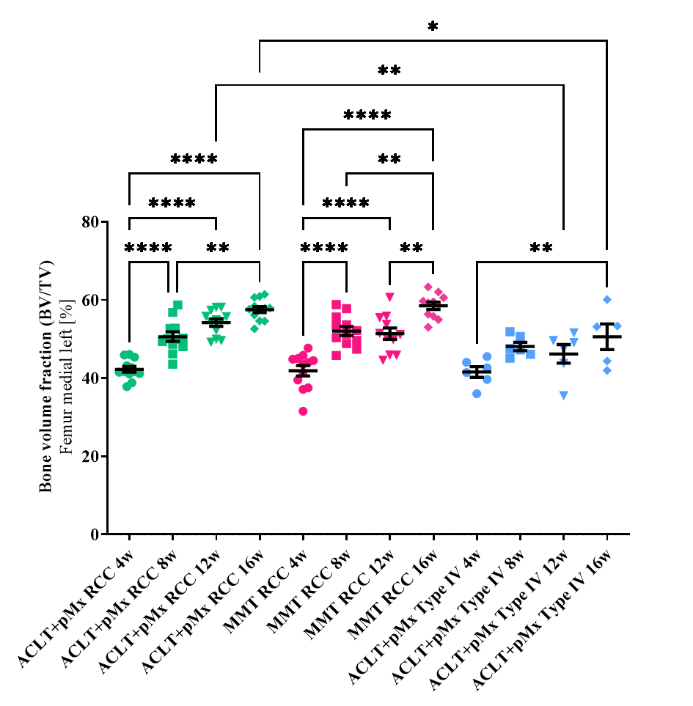


**Supplementary Figure 5.** Bone volume fraction analysis (BV/TV) of the left medial Femur; *n*=11/12 (RCC), *n*=5/6 (Type IV); mean ± SEM; no outliers via ROUT method detected; data passed Shaprio-Wilk test for normal distribution; results of 1way ANOVA with Šídák's multiple comparisons test: **p*<0.05 ACLT+pMx RCC 16w vs. ACLT+pMx Type IV 16w; ***p*<0.01 ACLT+pMx RCC 8w vs. ACLT+pMx RCC 16w; ***p*<0.01 MMT RCC 8w vs. MMT RCC 16w; ***p*<0.01 MMT RCC 12w vs. MMT RCC 16w; ***p*<0.01 ACLT+pMx Type IV 4w vs. ACLT+pMx Type IV 16w; ***p*<0.01 ACLT+pMx RCC 12w vs. ACLT+pMx Type IV 12w; *****p*<0.0001 ACLT+pMx RCC 4w vs. ACLT+pMx RCC 8w; *****p*<0.0001 ACLT+pMx RCC 4w vs. ACLT+pMx RCC 12w; *****p*<0.0001 ACLT+pMx RCC 4w vs. ACLT+pMx RCC 16w; *****p*<0.0001 MMT RCC 4w vs. MMT RCC 8w; *****p*<0.0001 MMT RCC 4w vs. MMT RCC 12w; *****p*<0.0001 MMT RCC 4w vs. MMT RCC 16w.


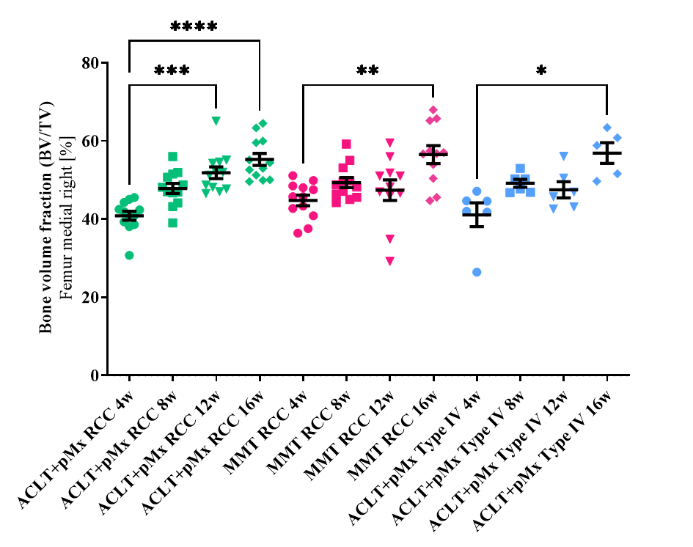


**Supplementary Figure 6.** Bone volume fraction analysis (BV/TV) of the right medial Femur; *n*=11/12 (RCC), *n*=5/6 (Type IV); mean ± SEM; no outliers via ROUT method detected; data did not pass Shaprio-Wilk test for normal distribution; results of Kruskal-Wallis test with Dunn’s multiple comparison test: **p*<0.05 ACLT+pMx Type IV 4w vs. ACLT+pMx Type IV 16w; ***p*<0.01 MMT RCC 4w vs. MMT RCC 16w; ****p*<0.001 ACLT+pMx RCC 4w vs. ACLT+pMx RCC 12w; *****p*<0.0001 ACLT+pMx RCC 4w vs. ACLT+pMx RCC 16w.


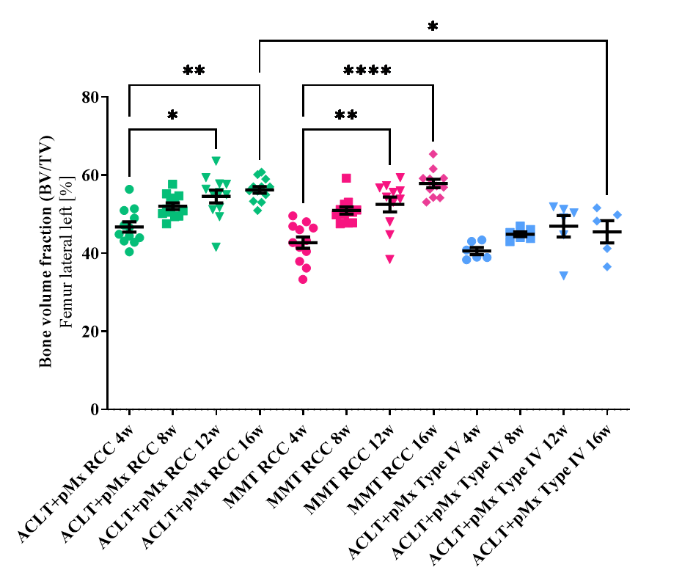


**Supplementary Figure 7.** Bone volume fraction analysis (BV/TV) of the left lateral Femur; *n*=11/12 (RCC), *n*=5/6 (Type IV); mean ± SEM; no outliers via ROUT method detected; data did not pass Shaprio-Wilk test for normal distribution; results of Kruskal-Wallis test with Dunn’s multiple comparison test: **p*<0.05 ACLT+pMx RCC 4w vs. ACLT+pMx RCC 12w; **p*<0.05 ACLT+pMx RCC 16w vs. ACLT+pMx Type IV 16w; ***p*<0.01 ACLT+pMx RCC 4w vs. ACLT+pMx RCC 16w; ***p*<0.01 MMT RCC 4w vs. MMT RCC 12w; *****p*<0.0001 MMT RCC 4w vs. MMT RCC 16w.


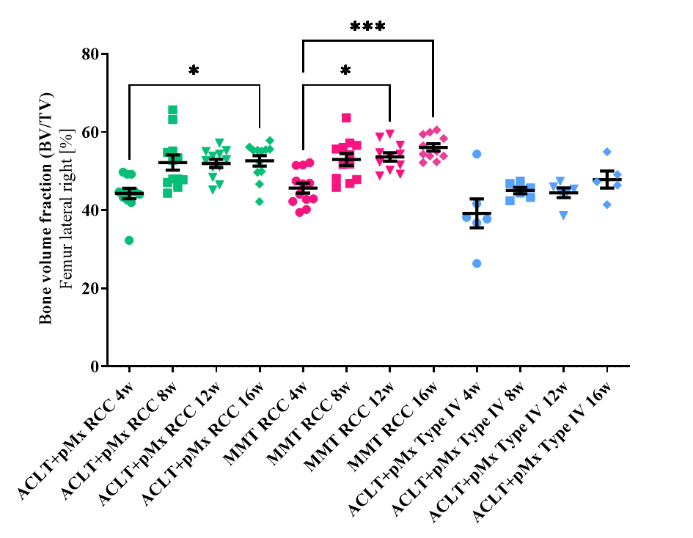


**Supplementary Figure 8.** Bone volume fraction analysis (BV/TV) of the right lateral Femur; *n*=11/12 (RCC), *n*=5/6 (Type IV); mean ± SEM; no outliers via ROUT method detected; data did not pass Shaprio-Wilk test for normal distribution; results of Kruskal-Wallis test with Dunn’s multiple comparison test: **p*<0.05 ACLT+pMx RCC 4w vs. ACLT+pMx RCC 16w; **p*<0.05 MMT RCC 4w vs. MMT RCC 12w; ****p*<0.001 MMT RCC 4w vs. MMT RCC 16w.


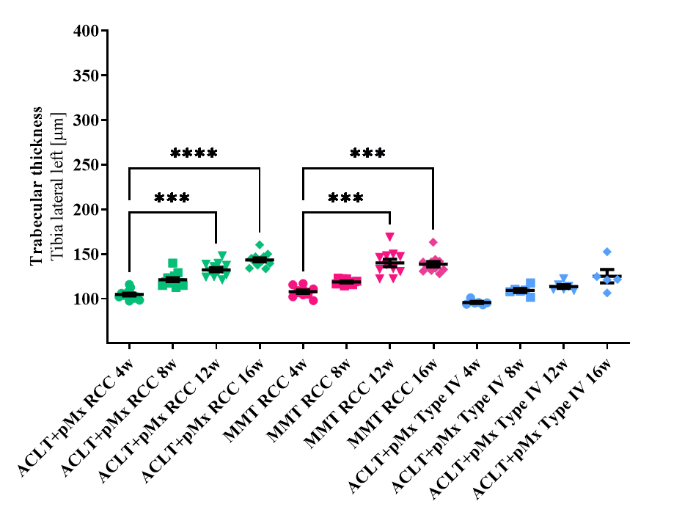


**Supplementary Figure 9.** Trabecular thickness (Tb.Th) of the left lateral Tibia; *n*=11/12 (RCC), *n*=5/6 (Type IV); mean ± SEM; two outliers via ROUT method detected and excluded (MMT RCC 4w: 178.05 µm; MMT RCC 8w: 142.68 µm); data did not pass Shaprio-Wilk test for normal distribution; results of Kruskal-Wallis test with Dunn’s multiple comparison test: ****p*<0.001 ACLT+pMx RCC 4w vs. ACLT+pMx RCC 12w; ****p*<0.001 MMT RCC 4w vs. MMT RCC 12w; *****p*<0.0001 ACLT+pMx RCC 4w vs. ACLT+pMx RCC 16w; *****p*<0.0001 MMT RCC 4w vs. MMT RCC 16w.


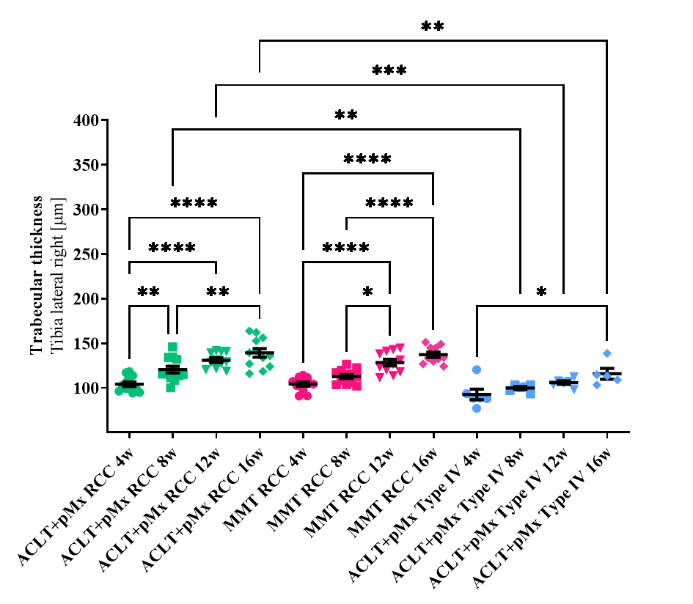


**Supplementary Figure 10.** Trabecular thickness (Tb.Th) of the right lateral Tibia; *n*=10-12 (RCC), *n*=5/6 (Type IV); mean ± SEM; two outliers via ROUT method detected and excluded (ACLT+pMx RCC 12w: 182.49 µm; MMT RCC 16w: 210.77 µm); data passed Shaprio-Wilk test for normal distribution; results of 1way ANOVA with Šídák's multiple comparisons test: **p*<0.05 MMT RCC 8w vs. MMT RCC 12w; **p*<0.05 ACLT+pMx Type IV 4w vs. ACLT+pMx Type IV 16w; ***p*<0.01 ACLT+pMx RCC 4w vs. ACLT+pMx RCC 8w; ***p*<0.01 ACLT+pMx RCC 8w vs. ACLT+pMx RCC 16w; ***p*<0.01 ACLT+pMx RCC 8w vs. ACLT+pMx Type IV 8w; ***p*<0.01 ACLT+pMx RCC 16w vs. ACLT+pMx Type IV 16w; ****p*<0.001 ACLT+pMx RCC 12w vs. ACLT+pMx Type IV 12w; *****p*<0.0001 ACLT+pMx RCC 4w vs. ACLT+pMx RCC 12w; *****p*<0.0001 ACLT+pMx RCC 4w vs. ACLT+pMx RCC 16w; *****p*<0.0001 MMT RCC 4w vs. MMT RCC 12w; *****p*<0.0001 MMT RCC 4w vs. MMT RCC 16w; *****p*<0.0001 MMT RCC 8w vs. MMT RCC 16w.


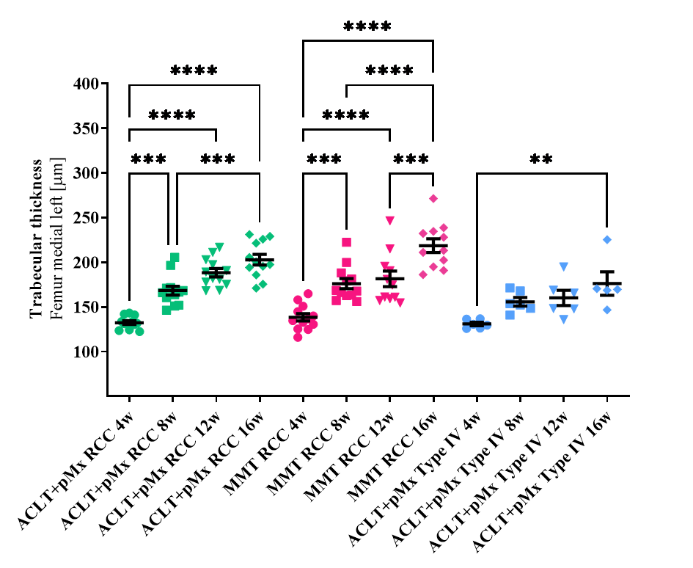


**Supplementary Figure 11.** Trabecular thickness (Tb.Th) of the left medial Femur; *n*=11/12 (RCC), *n*=5/6 (Type IV); mean ± SEM; no outliers via ROUT method detected; data passed Shaprio-Wilk test for normal distribution; results of 1way ANOVA with Šídák's multiple comparisons test: ***p*<0.01 ACLT+pMx Type IV 4w vs. ACLT+pMx Type IV 16w; ****p*<0.001 ACLT+pMx RCC 4w vs. ACLT+pMx RCC 8w; ****p*<0.001 ACLT+pMx RCC 8w vs. ACLT+pMx RCC 16w; ****p*<0.001 MMT RCC 4w vs. MMT RCC 8w; ****p*<0.001 MMT RCC 12w vs. MMT RCC 16w; *****p*<0.0001 ACLT+pMx RCC 4w vs. ACLT+pMx RCC 12w; *****p*<0.0001 ACLT+pMx RCC 4w vs. ACLT+pMx RCC 16w; *****p*<0.0001 MMT RCC 4w vs. MMT RCC 12w; *****p*<0.0001 MMT RCC 4w vs. MMT RCC 16w; *****p*<0.0001 MMT RCC 8w vs. MMT RCC 16w.


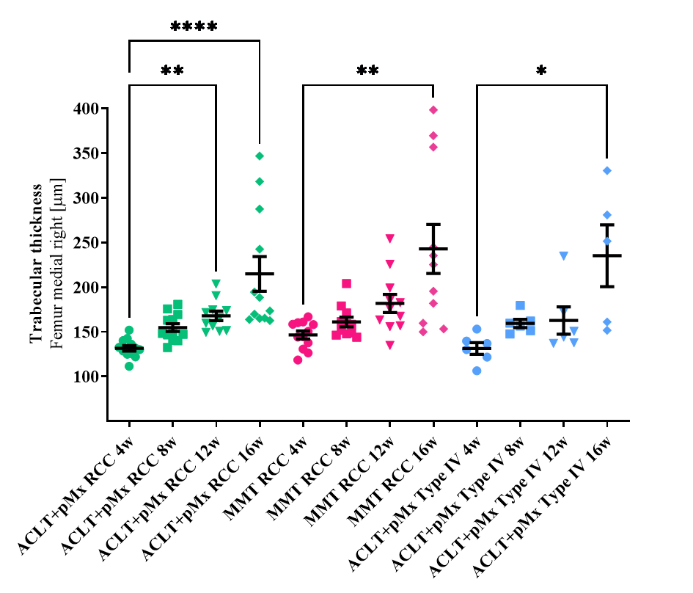


**Supplementary Figure 12.** Trabecular thickness (Tb.Th) of the right medial Femur; *n*=11/12 (RCC), *n*=5/6 (Type IV); mean ± SEM; two outliers via ROUT method detected and excluded (ACLT+pMx RCC 12w: 342.70 µm; MMT RCC 8w: 231.05 µm); data did not pass Shaprio-Wilk test for normal distribution; results of Kruskal-Wallis test with Dunn’s multiple comparison test: **p*<0.05 ACLT+pMx Type IV 4w vs. ACLT+pMx Type IV 16w; ***p*<0.01 ACLT+pMx RCC 4w vs. ACLT+pMx RCC 12w; ***p*<0.01 MMT RCC 4w vs. MMT RCC 16w; *****p*<0.0001 ACLT+pMx RCC 4w vs. ACLT+pMx RCC 16w.


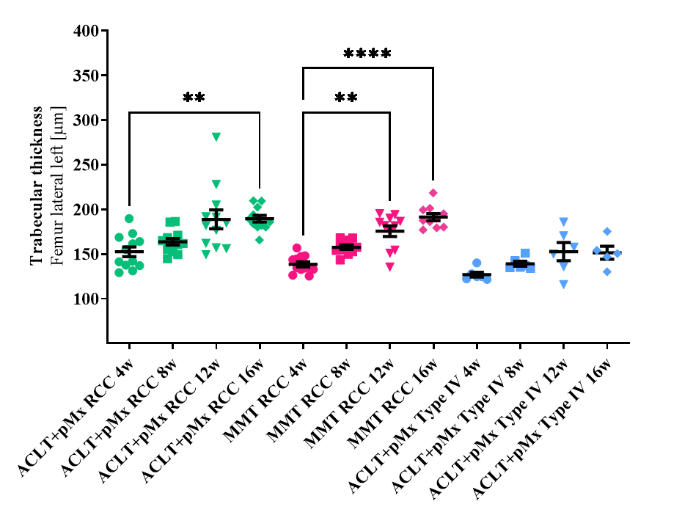


**Supplementary Figure 13.** Trabecular thickness (Tb.Th) of the left lateral Femur; *n*=10-12 (RCC), *n*=5/6 (Type IV); mean ± SEM; two outliers via ROUT method detected and excluded (MMT RCC 8w: 206.71 µm; MMT RCC 16w: 274.00 µm); data did not pass Shaprio-Wilk test for normal distribution; results of Kruskal-Wallis test with Dunn’s multiple comparison test: ***p*<0.01 ACLT+pMx RCC 4w vs. ACLT+pMx RCC 16w; ***p*<0.01 MMT RCC 4w vs. MMT RCC 12w; *****p*<0.0001 MMT RCC 4w vs. MMT RCC 16w.


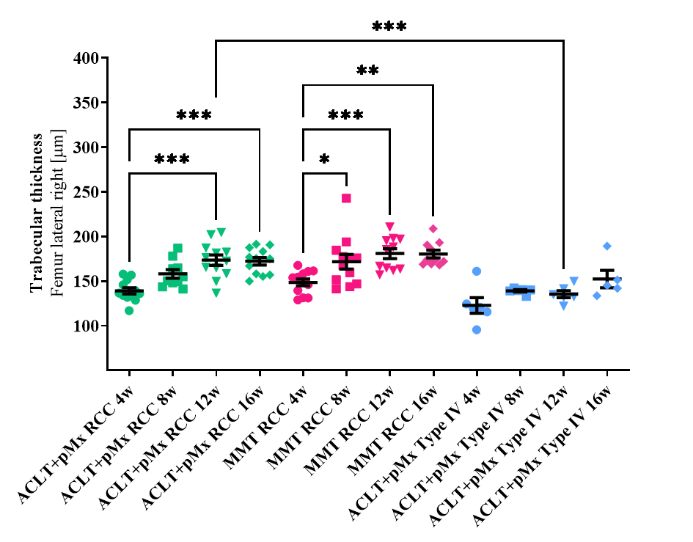


**Supplementary Figure 14.** Trabecular thickness (Tb.Th) of the right lateral Femur; *n*=10-12 (RCC), *n*=5/6 (Type IV); mean ± SEM; three outliers via ROUT method detected and excluded (ACLT+pMx RCC 8w: 284.10 µm, 225.86 µm; MMT RCC 16w: 263.71 µm); data passed Shaprio-Wilk test for normal distribution; results of 1way ANOVA with Šídák's multiple comparisons test: **p*<0.05 MMT RCC 4w vs. MMT RCC 8w; ***p*<0.01 MMT RCC 4w vs. MMT RCC 16w; ****p*<0.001ACLT+pMx RCC 4w vs. ACLT+pMx RCC 12w; ****p*<0.001 ACLT+pMx RCC 4w vs. ACLT+pMx RCC 16w; ****p*<0.001 MMT RCC 4w vs. MMT RCC 12w; ****p*<0.001 ACLT+pMx RCC 12w vs. ACLT+pMx Type IV 12w.


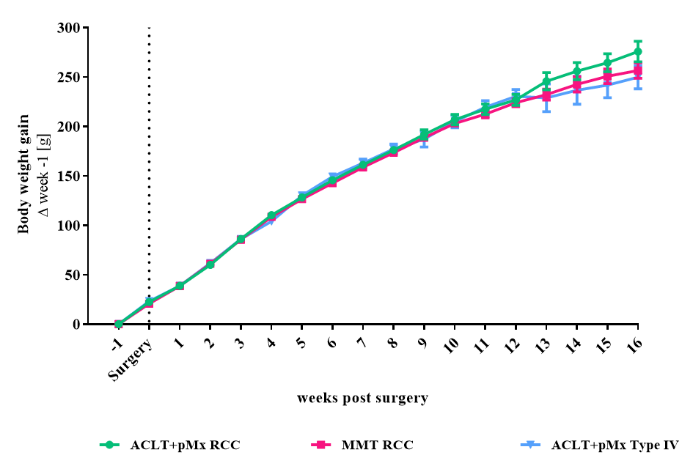


**Supplementary Figure 15.** Body weight gain over time; *n*=5-48; mean ± SEM; no outliers via ROUT method detected; data passed Shaprio-Wilk test for normal distribution; no significant differences in mixed-effects model analysis with Šídák's multiple comparisons test.
